# Soluble Form of Canine Transferrin Receptor Inhibits Canine Parvovirus Infection *In Vitro* and *In Vivo*

**DOI:** 10.1155/2013/172479

**Published:** 2013-09-08

**Authors:** Jiexia Wen, Sumin Pan, Shuang Liang, Zhenyu Zhong, Ying He, Hongyu Lin, Wenyan Li, Liyue Wang, Xiujin Li, Fei Zhong

**Affiliations:** ^1^Laboratory of Molecular Virology and Immunology, College of Veterinary Medicine, Agricultural University of Hebei, Baodiing 071001, China; ^3^Laboratory of Virology, College of Animal Science, Hebei Normal University of Science and Technology, Qinhuangdao 066600, China; ^2^Hebei Engineering and Technology Research Center of Veterinary Biological Products, North China Research Center of Animal Epidemic Pathogen Biology, China Agriculture Ministry, Baoding 071001, China; ^4^Cardinal Bernardin Cancer Center, Stritch School of Medicine, Loyola University Chicago, Maywood, IL 60153, USA; ^5^Department of Microbiology and Immunology, Stritch School of Medicine, Loyola University Chicago, Maywood, IL 60153, USA; ^6^Department of Biotechnology, College of Environmental and Chemical Engineering, Yanshan University, Qinhuangdao 066004, China

## Abstract

Canine parvovirus (CPV) disease is an acute, highly infectious disease threatening the dog-raising industry. So far there are no effective therapeutic strategies to control this disease. Although the canine transferrin receptor (TfR) was identified as a receptor for CPV infection, whether extracellular domain of TfR (called soluble TfR (sTfR)) possesses anti-CPV activities remains elusive. Here, we used the recombinant sTfR prepared from HEK293T cells with codon-optimized gene structure to investigate its anti-CPV activity both *in vitro* and *in vivo*. Our results indicated that codon optimization could significantly improve sTfR expression in HEK293T cells. The prepared recombinant sTfR possessed a binding activity to both CPV and CPV VP2 capsid proteins and significantly inhibited CPV infection of cultured feline F81 cells and decreased the mortality of CPV-infected dogs, which indicates that the sTfR has the anti-CPV activity both *in vitro* and *in vivo*.

## 1. Introduction

Canine parvovirus (CPV) was first identified in 1978 as a pathogen that is responsible for serious gastroenteritis and myocarditis in dogs [1]. Currently, there are mainly three antigenic types of CPV (CPV-2a, CPV-2b, and CPV-2c) that are prevalent in different countries and cause major infectious disease threatening dog-raising industry. CPV is a small nonenveloped, single-stranded DNA virus [2–4]. The linear single-strand DNA genome, about 5 kb long, contains two open reading frames (ORFs). One ORF encodes two nonstructural proteins (NS1 and NS2), while the other encodes two structural proteins (VP1 and VP2). VP2 (426 amino acid residues) is the main component in CPV capsid which possesses strong antigenic properties. The canine transferrin receptor (TfR) has been identified as a receptor for CPV infection mediated by VP2-TfR interaction [5–7]. 

TfR is a type II membrane glycoprotein expressed on the cell surface as a homodimer linked by two intermolecular disulphide bonds. Each monomer is comprised of extracellular domain, transmembrane domain, and intracellular domain. The extracellular domain includes stem, protease-like, apical, and helical regions [8]. Two disulphide bonds are located in the stem region. Under normal physiological conditions, TfR is involved in plasma-to-cell iron transport, in which iron-load transferrin (Tf) can specifically bind to TfR at the helical regions of TfR extracellular domains to form TfR-Tf complex and then enters the cell by endocytosis [9]. Meanwhile, CPV can also bind to TfR at the apical region of TfR extracellular domains through CPV VP2 and then infects host cells by endocytosis [7]. Therefore, both CPV and iron-loaded transferrin can bind to TfR; however, their binding sites are different. 

The viral receptor plays an important role in virus infection since it determines the species specificity, tissue tropism, and pathogenesis of the virus [10]. The virus infection is mediated by virus-receptor interaction. Blocking virus-receptor interaction is a common strategy for viral disease control [11–13]. Therefore, many antiviral drugs have been developed based on blocking virus-receptor interaction [14].

There are many strategies to block the virus-receptor interaction. One of most extensively used strategies in antiviral research and application is antibodies, which can specifically target the surface proteins of the viral particles, such as the spike proteins of enveloped viruses and the capsid proteins of nonenveloped viruses. These proteins on the viral surface are involved in viral attachment and infection to the host cells. Upon binding to viral surface proteins, the antibodies block the virus-receptor interaction and inhibit virus infection. For instance, the specific antibody against S2 domain of SARS spike protein could block SARS infection [15]. However, since the antibodies used to block virus infection mainly are polyclonal antibodies or anti-sera, which likely lead to animal infection caused by other pathogens contaminated in anti-sera. 

Another strategy for blocking the virus-receptor interaction is to use the small synthetic compounds obtained either by high-throughput screening of large antiviral compound libraries or by structure-guided rational design based on the structures and the interaction of viral surface proteins and receptors [16, 17]. Although small synthetic compounds may exhibit the potent antiviral activity, they may also cause some side effects. 

Using recombinant virus receptor to block the interaction between virus and host cells is another strategy for blocking the virus-receptor interaction [13]. For instance, Deen and colleagues have investigated using soluble form of recombinant CD4 protein to inhibit HIV virus infection [18], resulting in a significant inhibition of virus infectivity. 

Based on the interaction between CPV and TfR, we proposed that canine extracellular domain of TfR, also called soluble TfR (sTfR), might have the anti-CPV activities. To verify our hypothesis, we used the recombinant sTfR prepared in eukaryotic expression system with codon-optimized sTfR gene to test the anti-CPV of sTfR in cultured cells and in dogs. The results showed that the recombinant sTfR possessed significant anti-CPV activity.

## 2. Materials and Methods

### 2.1. Virus, Cell Lines, and Dogs

CPV-2a strain was a generous gift from Dr. Weiquan Liu (China Agricultural University). Feline kidney F81 cells used for propagation of CPV and human embryonic kidney (HEK) 293T cells used for expression of the recombinant sTfR were from China Center for Type Culture Collection in Beijing. The F81 cells or HEK293T cells were cultured in DMEM medium supplemented with 10% (v/v) FBS, 100 U/mL penicillin, 100 *μ*g/mL streptomycin, 2 mM L-glutamine at 37°C and 5% CO_2_. The local healthy mongrel dogs (6–8 weeks old, 3-4 kg, unvaccinated, anti-CPV antibody negative (hemagglutination inhibition (HI) titer is below 1 : 20) used for sTfR anti-CPV assay *in vivo* were purchased from a local dog breeder. Dog experiments were performed according to the guide of Beijing Municipality on the Review of Welfare and Ethics of Laboratory Animals approved by the Beijing Municipality Administration Office of Laboratory Animals. 

### 2.2. Reagents

Mouse anti-His-tag monoclonal antibody and goat anti-mouse IgG-AP antibody (Santa Cruz Biotechnology, USA) were used for detection of recombinant sTfR by Western blot. Anti-CPV VP2 polyclonal antibody was prepared from a New Zealand White Rabbit immunized with recombinant VP2. The recombinant VP2 was prepared in our lab from Chinese hamster ovary (CHO) cell line stably expressing VP2 [19, 20]. pcDNA3.1-CD5 plasmids containing human CD5 signal peptide were constructed as previously described [20]. Ni-NTA-agarose beads (Qiagen, Germany) were used for sTfR purification. Prestained protein marker and Bio-Rad protein assay kit were purchased from Bio-Rad Laboratories. Dulbecco's modified Eagle's medium (DMEM) and fetal bovine serum (FBS) were purchased from Gibco BRL Life Technologies. 

### 2.3. Codon Optimization and sTfR Expression Vector Construction

The codons for sTfR were optimized using human preferred codons based on Codon Usage Database (Kazusa DNA Research Institute, Japan). The codon-optimized sTfR gene was synthesized by Shanghai Sangon Biological Engineering Technology & Services. *Nhe*I and *Age*I restriction sites were introduced at 5′ terminus and 3′ terminus of the sTfR sequence, respectively. The wild-type sTfR gene was amplified from canine liver based on GenBank sequence (NM_001003111) by RT-PCR using a pair of primers: 5′cgc*gctagc*(*Nhe*I)*c*ggctactgt aaacgtgtagaac, 5′ccg*accggt*(*Age*I)aaactcattgtcaatatcccat. The wild-type and codon-optimized sTfR genes were subcloned into pcDNA3.1A-CD5 vector to construct their eukaryotic secretory expression vectors, pcDNA3.1A-CD5-sTfRw/His and pcDNA3.1A-CD5-sTfRopt/His, respectively. 

### 2.4. Transfection and Expression

The transfections in HEK293T cells were performed using modified calcium phosphate transfection method as previously described [21].

### 2.5. Recombinant sTfR Protein Purification

For sTfR purification, the transiently expressed sTfR in HEK293T cell culture medium was absorbed with Ni-NTA agarose beads (0.5 mL Ni-NTA slurry for 15 mL medium) and incubated at 4°C overnight with shaking to immobilize His-tag fused sTfR. After precipitation, the beads were washed four times with washing buffer (20 mM Tris, 500 mM NaCl, 10 mM imidazole, pH 8.0). Bound sTfR was eluted with elution buffer (20 mM Tris, 500 mM NaCl, 250 mM imidazole, pH 8.0). The eluted sTfR was dialyzed against PBS (pH 8.0) to eliminate imidazole. The protein concentrations were determined using Bradford method. 

### 2.6. SDS-PAGE and Western Blot

The protein sample was subjected to sodium dodecyl sulfate polyacrylamide gel electrophoresis (SDS-PAGE) on 10% (w/v) gels. The separated proteins on the gel were either stained with Coomassie Brilliant Blue or electroblotted onto a nitrocellulose membrane by using semidry electroblotting apparatus (Bio-Rad). Blotted proteins on the membrane were detected using a mouse anti-His-tag monoclonal antibody followed by a goat anti-mouse IgG conjugated with alkaline phosphatase. The blots were developed using BCIP/NBT substrate system (Bio-Rad).

### 2.7. sTfR Binding Assay

A slightly modified enzyme-linked immunosorbent assay (ELISA), as previously described [22], was used for detection of sTfR binding activities to VP2 proteins and CPV. Briefly, the 96-well plate was coated with VP2 (1 *μ*g/mL) and CPV (1 × 10^5^ TCID_50_/mL) diluted with coat buffer (0.1 M sodium carbonate buffer, pH 9.6), respectively, and blocked with 5% (w/v) nonfat milk in PBST. The rabbit anti-VP2 polyclonal antibodies (1 *μ*g/mL) were added into the VP2- or CPV-coated wells for antibody blocking test. After washing, the different amounts of sTfR (5, 2.5, 1.25, 0.63, 0.31, and 0.16 *μ*g/mL) in PBST were added into the well for sTfR binding to CPV or VP2 protein. The same amount of BSA was used as a negative control. The mouse anti-His-tag antibody (1 : 1000) and goat anti-mouse IgG-AP (1 : 1000) were used for detection of the adsorbed sTfR fused with His-tag. After development with 4-nitrophenyl phosphate solution, the optical density (*OD*) of each well was measured with a microplate reader at 405 nm. The sTfR binding activity was indicated by *OD* values.

### 2.8. Antiviral Assay of sTfR *In Vitro*

For antiviral assay* in vitro*, the feline kidney F81 cells were cultured in DMEM complete medium. When cells reached 95% of confluence, the cells were trypsinized and seeded into a 96-well plate at the density of 10^4^ cells/well. At the same time, 10 *μ*L CPV with the titer of 1 × 10^5^ TCID_50_/mL was mixed with 40 *μ*L of 0.1 *μ*g/*μ*L TfR protein or with 40 *μ*L of 0.1 *μ*g/*μ*L BSA as a negative control and incubated at room temperature for 30 min. 50 *μ*L of the mixture above was added into each cell and incubated in 5% CO_2_ at 37°C for 6 h. The cell medium was replaced with DMEM complete medium containing 10% FBS and antibiotics and continued to be cultured for 36~48 h. The cell morphology was checked at the different time points during the culture period using light microscopy. When the negative control cells (treated with BSA + CPV-2a) show obviously pathologic changes, the cells were harvested and were subject to repeated freezing/thawing three times. CPV titer for each well was measured by TICD_50_ assay. The anti-CPV activity of recombinant sTfR was evaluated by the viral titer reduction in feline F81 cells. 

### 2.9. Antiviral Assay of sTfR *In Vivo*

To evaluate sTfR antiviral activity *in vivo*, 24 local mongrel dogs (6–8 weeks old, 3-4 kg, unimmunized and anti-CPV antibody negative, screened by ELISA with the recombinant CPVVP2) were divided into 3 groups. Eight dogs in group 1 were preinjected intravenously with sTfR (20 mg/kg) at 24 h before virus challenge. Then, all dogs in the three groups were challenged with CPV-2a (5 × 10^7^ TICD_50_/kg) by oral infection. The dogs in group 2 (8 dogs) were treated with sTfR with 20 mg/kg doses at 24 h after virus challenge. The dogs in group 3 as a negative control were treated with BSA at the same time point. The dogs in group 1 and group 2 were continuously treated with sTfR at 10 mg/kg/day doses. At the 10 days after challenge, the morbidity and mortality rates of infected dogs in all groups were calculated. The antiviral activity of the sTfR *in vivo *was evaluated by the reductions of dog morbidity and mortality rates.

### 2.10. Amplification of CPV and Virus Titer Determination

CPV was amplified in F81 cells and the virus titers determined by TCID_50_.

### 2.11. Statistical Analysis

Data were analyzed with SPSS 18.0, and the statistically significant differences were checked by *t* test.

## 3. Results

### 3.1. Construction of sTfR Expression Vector and Its Secretory Expression in HEK293T Cells

**Figure 1:**
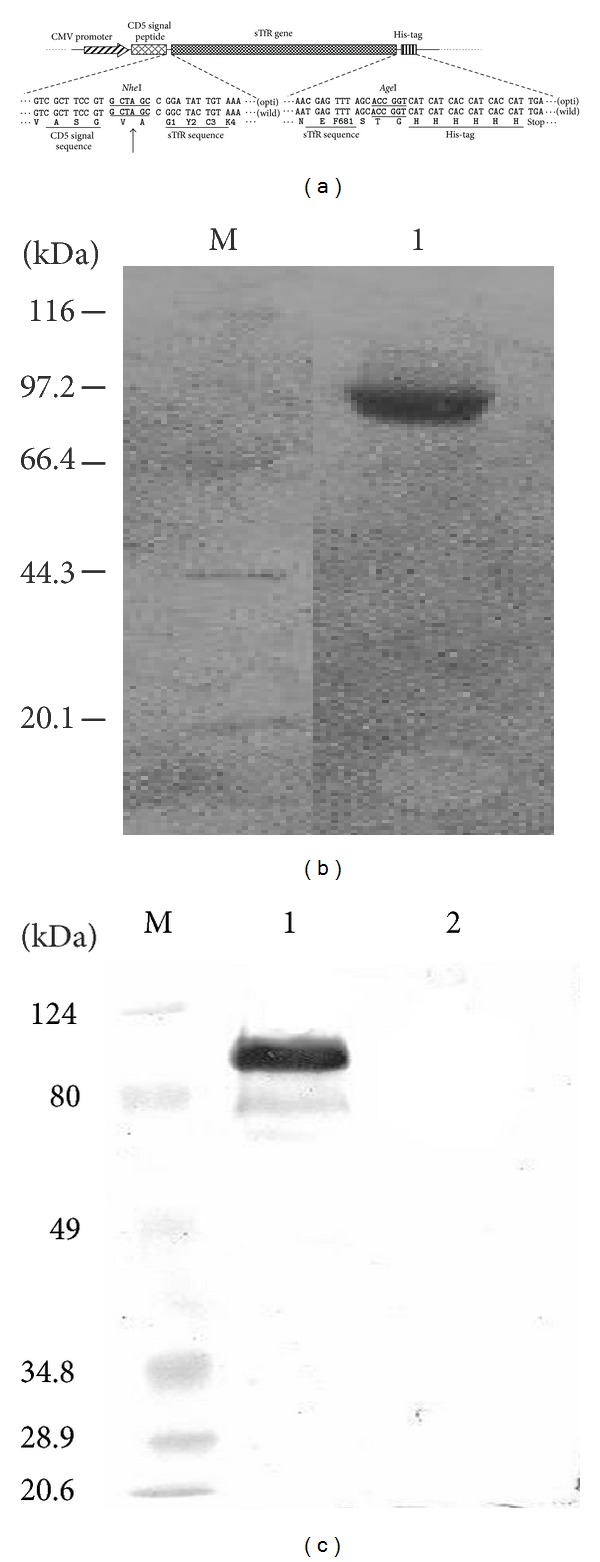
Structures of sTfR expression vectors and identification of sTfR expression. (a) The wild-type or codon-optimized sTfR gene expression vectors, pcDNA3.1A-CD5-sTfRw/His and pcDNA3.1A-CD5-sTfRopt/His, in which sTfR gene driven by CMV promoter was fused by human CD5 signal peptide sequence at 5′-terminus and 6 × His-tag at 3′-terminus. (b) Identification of sTfR with SDS-PAGE: lane M, protein markers; lane 1, purified sTfR. (c) Identification of sTfR with Western blot: lane M, prestained protein markers; lane 1, sTfR protein expressed from pcDNA3.1A-CD5-sTfRopt/His-transfected cells; lane 2, negative control from empty vector-transfected cells.

Canine TfR is a glycoprotein. To prepare biologically active sTfR, the eukaryotic cell line, HEK293T cells were used. First the wild-type sTfR gene (encoding amino acid sequence: G_1_W_2_C_3_K_4_ *⋯⋯*N_679_E_680_F_681_) was amplified by RT-PCR and sTfR codons were optimized with human preferred codons. Wild-type and codon-optimized sTfR gene expression vectors (pcDNA3.1A-CD5-sTfRw/His and pcDNA3.1A-CD5-sTfRopt/His) were constructed using pcDNA3.1A-CD5 vector (Figure 1(a)), in which the sTfR genes were driven by CMV promoter and fused with human CD5 leader sequence at the N-terminus and His-tag at the C-terminus: the former mediates sTfR secretion, and the later facilitates sTfR protein purification.

To prepare recombinant sTfR, the sTfR vectors, either wild-type or codon-optimized, were transfected into HEK293T cells with calcium phosphate transfection method (pcDNA3.1-CD5 empty vector as a negative control). At 48 h posttransfection, the culture medium was harvested and the recombinant sTfR was purified by Ni-NTA agarose and identified by SDS-PAGE (Figure 1(b)) and Western blot (Figure 1(c)). 

As shown in Figures 1(b) and 1(c), about 90 kDa protein bands were detected either by SDS-PAGE or Western blot (Figure 1(b), lane 1, and Figure 1(c), lane 1), a little higher than the calculated sTfR molecular weight (80 kDa), which might be caused by glycosylation. Those results indicated that the recombinant sTfR could be expressed in HEK293T cells and secreted into the culture medium. 

### 3.2. Codon Optimization Can Increase sTfR Expression in Eukaryotic Cells

**Figure 2:**
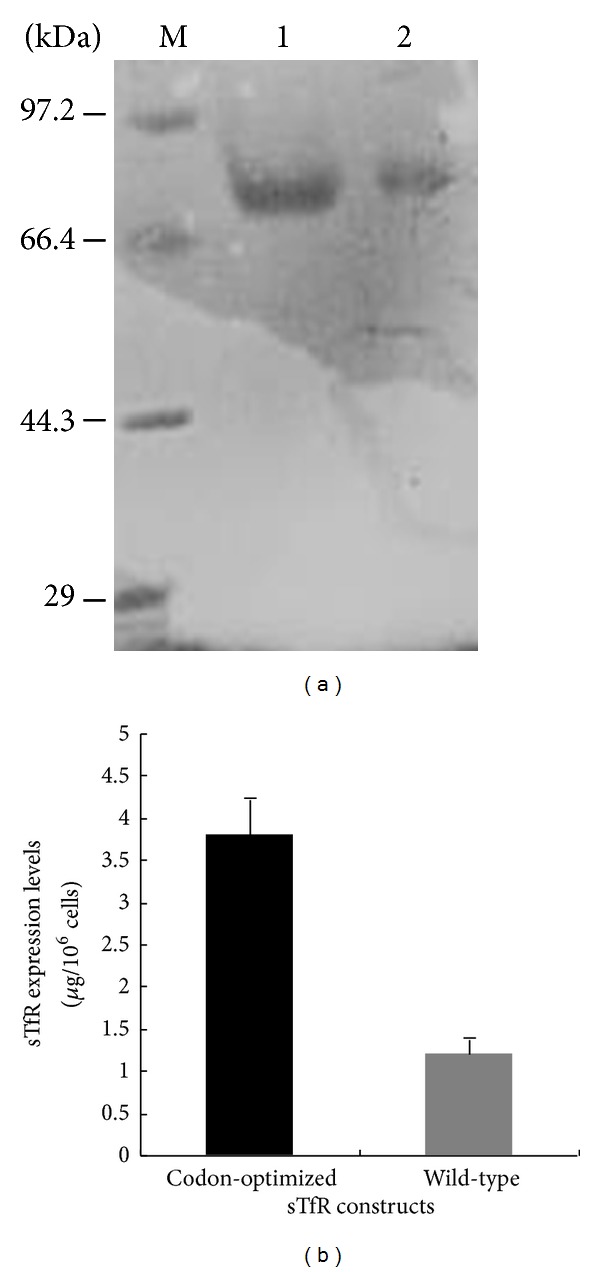
The effect of codon optimization on sTfR expression. (a) SDS-PAGE analysis for purified sTfR proteins. Lane 1, sTfR protein expressed with codon-optimized construct; lane 2, sTfR protein expressed with wild-type construct; M, protein marker. (b) Comparison of the protein expression levels with the different sTfR gene constructs.

To evaluate the effect of codon optimization on sTfR expression in eukaryotic cells, we compared the expression levels of wild-type and codon-optimized sTfR genes in HEK293T cells. It can be seen from Figure 2 that the expression level of codon-optimized sTfR gene (3.7 ± 0.21 *μ*g/10^6^ cells) was 3-fold higher than that of the wild-type gene (1.2 ± 0.12 *μ*g/10^6^ cells), indicating that codon optimization could significantly increase sTfR expression in eukaryotic cells.

### 3.3. sTfR Can Bind to CPV and CPV VP2

**Figure 3:**
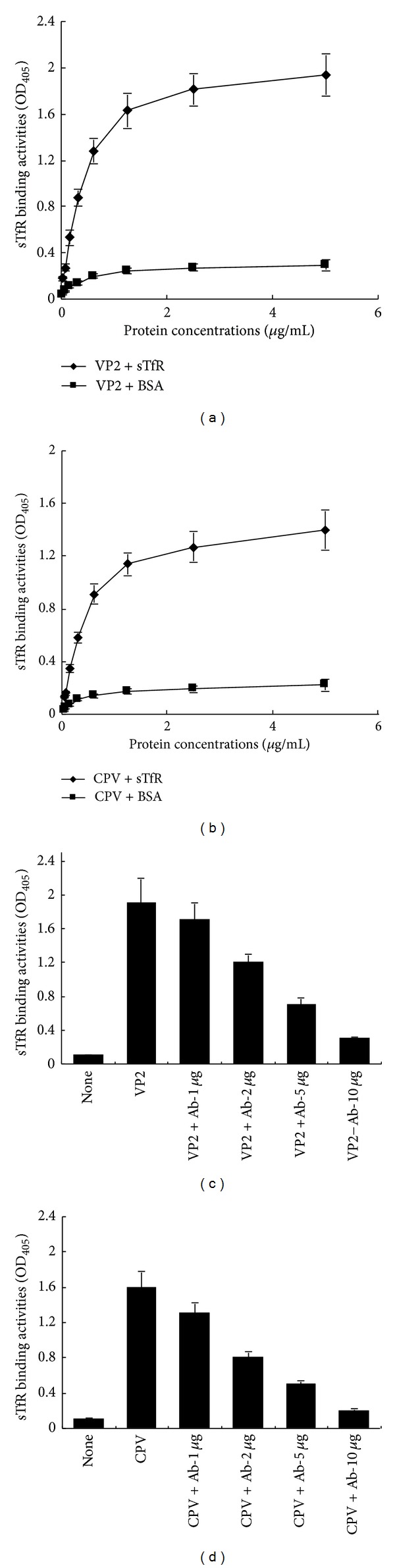
The recombinant sTfR binding activities to VP2 and CPV. Two 96-well plates were coated with VP2 (1 *μ*g/mL) (a) and CPV (1 × 10^−5^ TCID_50_/mL) (b), respectively. The different amounts of sTfR (5, 2.5, 1.25, 0.63, 0.31, and 0.16 *μ*g/mL) were added for each well (BSA as a negative control). Other two 96-well plates were also coated with VP2 (1 *μ*g/mL) (c) and CPV (1 × 10^−5^ TCID_50_/mL) (d) and were added rabbit anti-VP2 polyclonal antibodies at different amount (1, 2, 5, 10 *μ*g/mL) for antibody blocking test. Then the TfR (2.5 *μ*g/mL) was added for all of the wells except mock control well. The mouse anti-His antibody and goat anti-mouse IgG-AP were used for detection of the bound sTfR fused with His-tag. After development with 4-nitrophenyl phosphate substrate, the optical density (*OD*) for each well was measured using microplate reader at 405 nm. The sTfR binding activity was indicated by *OD* values.

The VP2 protein has been identified to interact with canine TfR during CPV attachment and infection to the host cells [6]. To investigate whether the recombinant canine sTfR has the ability to bind to CPV and VP2, we used CPV-2a strain and the recombinant VP2 (prepared in our lab) to test sTfR binding activity using ELISA method. Binding kinetics (Figures 3(a) and 3(b)) showed that sTfR could bind to both VP2 and CPV in a dose-dependent manner. This binding process could be blocked by VP2 antibody, and the blocking capability was directly proportional to the dosages of VP2 antibody (Figures 3(c) and 3(d)). These results indicate that the recombinant sTfR prepared in this study can bind to CPV and VP2. 

### 3.4. sTfR Inhibits CPV Infection to Host Cells

**Figure 4:**
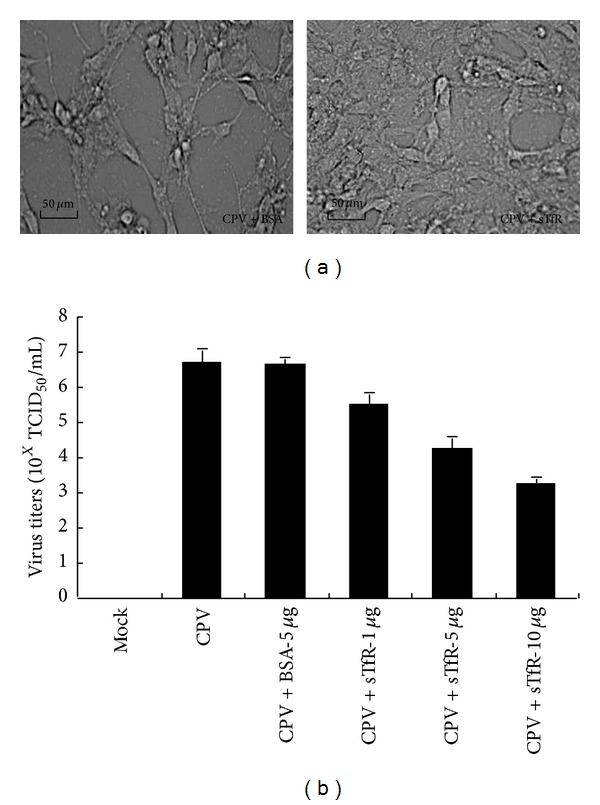
Effects of sTfR on F81 cell morphology and viral tilters after CPV infection. (a) Effects of sTfR on F81 cell morphology: CPV (1 × 10^5^ TCID_50_/mL) was incubated with same volume of 10 *μ*g/mL sTfR or 10 *μ*g/mL BSA (control). F81 cells seeded into 96-well plate at the density of 1 × 10^5^ cells/mL (50 *μ*L). The sTfR-incubated or BSA-incubated (as a control) CPV was added to the well and incubated at 37°C for 6 h. Unbound viruses were removed by extensive washing with DMEM. The cells were continuously cultured until CPE occurred.  Figure 4(a) shows the morphology of F81 cells infected with BSA-incubated CPV (left) and sTfR-incubated CPV (right) at 48 h postinfection, respectively. (b) Effects of sTfR on CPV titer in F81 cells: F81 cells were infected by CPV preincubated with different amounts of sTfR and BSA. The infected cells were harvested at 48 h postinfection. The virus particles were released from the cells by the repeated freezing/shawing. The virus titer was estimated by TCID_50_.

To identify whether recombinant sTfR has the ability to inhibit CPV infection, CPV-2a strain was incubated with sTfR or BSA (as a negative control) at the ratio of 1 : 500 (1 viral particle versus 500 sTfR molecules) at 37°C for 1 h. The host cells F81 were then infected with the sTfR-incubated CPV-2a in a 96-well plate. The morphology of F81 cells was examined at different time points after the infection. CPV viral titer was calculated with TCID_50_.  Figure 4(a) showed the morphology of the CPV-infected cells at 48 h postinfection.  Figure 4(b) showed the CPV titers in F81 cells treated with different dosages of sTfR. 

As shown in Figure 4(a), the cytopathic effect (CPE) of F81 cells treated with CPV/BSA was much more obvious than that of the cells treated with sTfR/CPV at 48 h postinfection. The CPV titer in sTfR/CPV-infected F81 cells (10^3.26^ TCID_50_/mL) was significantly reduced compared with that of the CPV/BSA-infected cells (10^6.67^ TCID_50_/mL) or CPV-infected cells (10^6.70^ TCID_50_/mL) (Figure 4(b)). The degree of virus reduction is proportional to the dosages of sTfR. The above results indicate that the recombinant sTfR can significantly inhibit CPV infection to the host cells* in vitro*.

### 3.5. sTfR Decreases Mortality Rates of CPV-Infected Dogs

**Figure 5:**
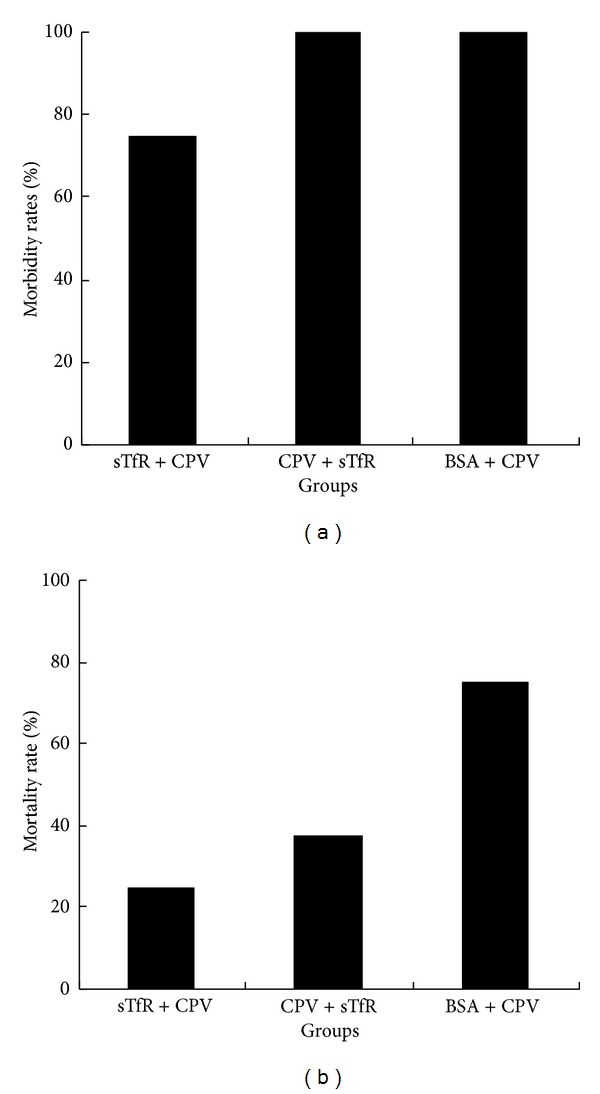
The effects of sTfR treatments on the morbidity (a) and mortality (b) rates of CPV-infected dogs. The dogs were treated with sTfR before CPV challenge (sTfR + CPV) and after CPV challenge (CPV + sTfR). CPV + BSA indicates the control without sTfR treatment.

To evaluate sTfR anti-CPV activity *in vivo*, the dogs were divided into three groups. Group 1 was treated with sTfR before CPV challenge, while group 2 was treated with sTfR after CPV challenge. Group 3, as a negative control group, was treated with BSA before CPV challenge. Ten days post-challenge, the morbidity and mortality rates of CPV-infected dogs in the three groups were calculated (Figure 5). The mortality rates (Figure 5(b)) of CPV-infected dogs either pretreated (sTfR + CPV) or post-treated (CPV + sTfR) with sTfR tended to be lower than that of the control (CPV + BSA). Notably, the mortality rates of CPV-infected dogs in pretreated (sTfR + CPV) group decreased more than 2 times compared with the control group. Interestingly, the mortality (Figure 5(b)) rates of sTfR-pretreated dogs were lower than that of sTfR-posttreated dogs, suggesting that sTfR shows not only the therapeutic effects, but also preventive effects. Our results preliminarily indicate that sTfR tends to inhibit CPV infection *in vivo*. 

## 4. Discussion 

In the present study, we investigated whether recombinant sTfR has the ability to block the CPV infection. Our results show that sTfR could significantly inhibit the CPV infection to the host cells *in vitro* and tended to decrease the mortality of the experimentally CPV-infected dogs *in vivo*, suggesting that sTfR has the potential protective effect for CPV control, which provides an important clue for further developments of new anti-CPV agent based on sTfR. 

To prepare glycosylated and dimeric recombinant TfR, eukaryotic cell line (HEK293T) was used to express the sTfR for its posttranslational glycosylation and proper folding. The cysteine residue-containing (GY**C**_3_KRVEPKAG**C**_12_ER) fragment in the stem of TfR extracellular domain remained in sTfR gene construct for sTfR dimerization through two helix domains interaction and two intermolecular disulphide bounds formation. Our data showed that the sTfR prepared in this study could bind to CPV and VP2 with a high affinity, suggesting that the sTfR possesses the biological activity. We previously used *E. coli* expression system to prepare the recombinant sTfR; the resulting recombinant sTfR was unable to effectively bind to VP2 and CPV probably due to lacking posttranslational modifications, including glycosylation and disulphide formation because bacterial cytoplasm is a reducing condition that is not favorable for disulfide bound formation [23]. 

Codon optimization is a common strategy for the increase of recombinant protein expression. Potent signal peptide sequence could allow recombinant protein to be effectively expressed and secreted [21, 24]. By adopting the strategies of codon optimization and human CD5-signal-peptide mediation, the high-level secretory expression was obtained in this study, and the expression level reached more than 3 *μ*g/10^6^ cells, which lays the foundation for the large-scale preparation of recombinant sTfR for further application. 

In the present study, we have only analyzed the sTfR antiviral activity to the CPV-2a subtype, without testing CPV-2b and CPV-2c subtype. We speculated that the affinity of sTfR to the different subtypes of CPV might be different since the classification of CPV subtypes is based on VP2 gene sequence, and the infection of CPV to host cells is mediated by VP2-TfR interaction. Thus, the sTfR anti-CPV activity may be different against distinct subtypes of CPV. Nonetheless, it would be interesting and important to test the anti-CPV activity of sTfR for other subtypes of CPV in future studies.

Lastly, the interaction between the soluble receptor and its natural ligand should also be considered during the endeavors of developing soluble receptor-based antiviral agents because the recombinant receptor can bind both virus and natural ligand. The recombinant sTfR bind not only to CPV but also to transferrin, a natural ligand. Although the two ligands do not compete with each other to bind to the natural TfR because their binding sites on the extracellular domain of TfR are different [25], however, whether the recombinant sTfR interferes with the transferrin-TfR complex internalization, or vice versa, remains to be elucidated. Reducing the affinity of recombinant sTfR to the transferrin through gene mutagenesis would help to weaken the side effects.

## References

[1] Thomson GW, Gagnon AN (1978). Canine gastroenteritis associated with a parvovirus-like agent. *The Canadian Veterinary Journal*.

[2] Parrish CR, O’Connell PH, Evermann JF, Carmichael LE (1985). Natural variation of canine parvovirus. *Science*.

[3] Decaro N, Buonavoglia C (2012). Canine parvovirus—a review of epidemiological and diagnostic aspects, with emphasis on type 2c. *Veterinary Microbiology*.

[4] Hueffer K, Parker JSL, Weichert WS, Geisel RE, Sgro J-Y, Parrish CR (2003). The natural host range shift and subsequent evolution of canine parvovirus resulted from virus-specific binding to the canine transferrin receptor. *Journal of Virology*.

[5] Hueffer K, Govindasamy L, Agbandje-McKenna M, Parrish CR (2003). Combinations of two capsid regions controlling canine host range determine canine transferrin receptor binding by canine and feline parvoviruses. *Journal of Virology*.

[6] Parker JSL, Murphy WJ, Wang D, O’Brien SJ, Parrish CR (2001). Canine and feline parvoviruses can use human or feline transferrin receptors to bind, enter, and infect cells. *Journal of Virology*.

[7] Hueffer K, Palermo LM, Parrish CR (2004). Parvovirus infection of cells by using variants of the feline transferrin receptor altering clathrin-mediated endocytosis, membrane domain localization, and capsid-binding domains. *Journal of Virology*.

[8] Lawrence CM, Ray S, Babyonyshev M, Galluser R, Borhani DW, Harrison SC (1999). Crystal structure of the ectodomain of human transferrin receptor. *Science*.

[9] Luck AN, Mason AB (2012). Transferrin-mediated cellular iron delivery. *Current Topics in Membranes*.

[10] Ibricevic A, Pekosz A, Walter MJ (2006). Influenza virus receptor specificity and cell tropism in mouse and human airway epithelial cells. *Journal of Virology*.

[11] Vermeire K, Schols D, Bell TW (2006). Inhibitors of HIV infection via the cellular CD4 receptor. *Current Medicinal Chemistry*.

[12] Vermeire K, Schols D (2005). Anti-HIV agents targeting the interaction of gp120 with the cellular CD4 receptor. *Expert Opinion on Investigational Drugs*.

[13] Marlin SD, Staunton DE, Springer TA, Stratowa C, Sommergruber W, Merluzzi VJ (1990). A soluble form of intercellular adhesion molecule-1 inhibits rhinovirus infection. *Nature*.

[14] Pöhlmann S, Doms RW (2002). Evaluation of current approaches to inhibit HIV entry. *Current Drug Targets—Infectious Disorders*.

[15] Leung KM, Feng DX, Lou J (2008). Development of human single-chain antibodies against SARS-associated coronavirus. *Intervirology*.

[16] Che P, Wang L, Li Q (2009). The development, optimization and validation of an assay for high throughput antiviral drug screening against dengue virus. *International Journal of Clinical and Experimental Medicine*.

[17] Li Q, Maddox C, Rasmussen L, Hobrath JV, White LE (2009). Assay development and high-throughput antiviral drug screening against Bluetongue virus. *Antiviral Research*.

[18] Deen KC, McDougal JS, Inacker R (1988). A soluble form of CD4 (T4) protein inhibits AIDS virus infection. *Nature*.

[19] Wang W, Li X, Wang X, Han D, Pan S, Zhong F (2010). Establishment of CHO-K1 cell lines stably expressing canine parvovirus VP2 structure protein. *Chinese Journal of Veterinary Science*.

[20] Wang W, Li X, Zhong F (2009). Secreting expression and characterization of canine parvovirus VP2 protein in eukaryotic cells. *Wei Sheng Wu Xue Bao*.

[21] Jin H, Zhong F, Wu L, Xie M, Wang W, Han D (2008). Construction of eukaryotic expression vector carrying canine *β*-defensin-1 gene and its expression in HEK293T cells. *Current Zoology*.

[22] Pan S, Li X, Zhong F, Wang X, Han D, Wang W (2009). Study on codon optimization and expression of canine sTfR gene in eukaryotic cells and interaction with canine parvovirus VP2 protein. *Chinese Journal of Veterinary Science*.

[23] Derman AI, Prinz WA, Belin D, Beckwith J (1993). Mutations that allow disulfide bond formation in the cytoplasm of Escherichia coli. *Science*.

[24] Zakhartchouk AN, Viswanathan S, Moshynskyy I, Petric M, Babiuk LA (2007). Optimization of a DNA vaccine against SARS. *DNA and Cell Biology*.

[25] Palermo LM, Hueffer K, Parrish CR (2003). Residues in the apical domain of the feline and canine transferrin receptors control host-specific binding and cell infection of canine and feline parvoviruses. *Journal of Virology*.

